# The irregular firing properties of thalamic head direction cells mediate turn-specific modulation of the directional tuning curve

**DOI:** 10.1152/jn.00583.2013

**Published:** 2014-08-13

**Authors:** Marian Tsanov, Ehsan Chah, Muhammad S. Noor, Catriona Egan, Richard B. Reilly, John P. Aggleton, Jonathan T. Erichsen, Seralynne D. Vann, Shane M. O'Mara

**Affiliations:** ^1^Trinity College Institute of Neuroscience, Trinity College Dublin, Dublin, Ireland;; ^2^School of Psychology, Trinity College Dublin, Dublin, Ireland;; ^3^Trinity Centre for Bioengineering, Trinity College Dublin, Dublin, Ireland;; ^4^School of Psychology, Cardiff University, Cardiff, United Kingdom; and; ^5^School of Optometry and Vision Sciences, Cardiff University, Cardiff, United Kingdom

**Keywords:** head direction, anterior thalamus, high-voltage spindles, Hodgkin-Huxley model

## Abstract

Head direction cells encode an animal's heading in the horizontal plane. However, it is not clear why the directionality of a cell's mean firing rate differs for clockwise, compared with counterclockwise, head turns (this difference is known as the “separation angle”) in anterior thalamus. Here we investigated in freely behaving rats whether intrinsic neuronal firing properties are linked to this phenomenon. We found a positive correlation between the separation angle and the spiking variability of thalamic head direction cells. To test whether this link is driven by hyperpolarization-inducing currents, we investigated the effect of thalamic reticular inhibition during high-voltage spindles on directional spiking. While the selective directional firing of thalamic neurons was preserved, we found no evidence for entrainment of thalamic head direction cells by high-voltage spindle oscillations. We then examined the role of depolarization-inducing currents in the formation of separation angle. Using a single-compartment Hodgkin-Huxley model, we show that modeled neurons fire with higher frequencies during the ascending phase of sinusoidal current injection (mimicking the head direction tuning curve) when simulated with higher high-threshold calcium channel conductance. These findings demonstrate that the turn-specific encoding of directional signal strongly depends on the ability of thalamic neurons to fire irregularly in response to sinusoidal excitatory activation. Another crucial factor for inducing phase lead to sinusoidal current injection was the presence of spike-frequency adaptation current in the modeled neurons. Our data support a model in which intrinsic biophysical properties of thalamic neurons mediate the physiological encoding of directional information.

head direction (HD) cells in anterior thalamus express a wide range of interspike intervals (ISIs) (Taube 2010). Given the importance of HD encoding for spatial navigation in the environment, it is crucial to understand whether intrinsic biophysical properties mediate the shape of directional tuning curves. Here we ask how ISI patterns affect the formation of the HD tuning curve in the horizontal plane. ISI variability is evaluated by the coefficient of variation; high values reflect an irregular pattern of the spikes (Softky and Koch 1993). ISI variability is very sensitive to the mean firing rate (Holt et al. 1996; Softky and Koch 1993); however, large ISIs as part of intertrain analyses are not simply noise around the mean firing rate but reflect characteristics of the membrane potential (Angelo and Margrie 2011). To investigate the functional relation between ISI diversity and separation angle, we use a single-cell Hodgkin-Huxley-type model to simulate ISI patterns ranging from irregularly to regularly firing-type cells (Pospischil et al. 2008). We apply sinusoidal current injection that mimics the Gaussian distribution of HD signal in thalamic neurons (Taube et al. 1990). Experimental and theoretical evidence suggests that bursts occur preferentially on the ascending slope of input signal (Gabbiani et al. 1996; Guido et al. 1992; Kepecs et al. 2002). Thus irregularly spiking neurons may detect the rising slope of input signals with greater precision compared with regularly spiking neurons (Metzner et al. 1998; Sherman 2001).

Previous models of the anticipatory time interval (ATI; a parameter closely related to the separation angle) describe that directional anticipation relies on the firing properties of neurons afferent to the HD system (van der Meer et al. 2007). The idea that ATI actively predicts direction has been challenged (van der Meer et al. 2007), especially in the context of the finding that ATI does not represent motor efference copy (Bassett et al. 2005). ATI values differ substantially across sequentially connected regions, decreasing in the following order: mammillary bodies > anterior thalamus > postsubiculum (Blair et al. 1998; Blair and Sharp 1995; Stackman et al. 2003; Taube and Muller 1998). Compared with anterodorsal nucleus (AD) thalamic cells (∼25 ms), the ATI is greater for mammillary bodies (40–75 ms) but close to zero for the dorsal tegmental nucleus of Gudden (Sharp et al. 2001). The ATI in the retrosplenial cortex (∼25 ms) (Cho and Sharp 2001) is similar to thalamic values, although the postsubicular ATI differs substantially, with values close to 0. This raises the question of why the ATI differs so much in monosynaptically connected regions. Furthermore, it is unclear why ATI decreases but not increases with the propagation of the vestibular signal, which is proposed to follow a bottom-up direction (Taube 2007). We propose that turn-specific modulation of the tuning curve is not a process of anticipation but rather represents the intrinsic neuronal properties of the HD neurons. For this purpose, we evaluated the separation angle instead of ATI. Here we expand the investigation of separation angle formation to the ISI variability of thalamic HD cells. We recorded in AD and anteroventral (AV) thalamic nuclei to obtain higher variability in the ISIs of HD neurons. The AV HD population also expresses large ISIs within theta range (Tsanov et al. 2011a), which increase the coefficient of variation values. Based on experimental data, we use computational modeling to investigate how the irregularity of thalamic firing patterns reflects the susceptibility of thalamic neurons to turn-specific modulation of the HD tuning curve. We propose here that HD neurons, which express a higher degree of irregularity, fire more spikes in the ascending phase of the directional tuning curve, leading to higher clockwise (CW) versus counterclockwise (CCW) directional curve separation, compared with neurons that fire with a lower degree of irregularity.

One characteristic feature of anterior thalamic nuclei is that there is little evidence for local GABAergic interneurons (Wang et al. 1999). Computational network models of thalamic activity involving hyperpolarization of HD cells propose that inhibition in the anterior thalamus may depend on extrinsic circuits (Rubin et al. 2001). The anatomical substrate of thalamic inhibitory circuits is associated with the thalamic reticular nucleus, which provides a topographically organized GABAergic projection to the anterior thalamic nuclei (Gonzalo-Ruiz and Lieberman 1995; Lozsadi 1995). Importantly, the thalamic reticular nucleus (which is involved in the generation of spindles) evokes local field synchronization in anterior thalamus (Tsanov et al. 2011b). To test the hypothesis that rhythmic reticular inhibition evokes spiking activity of HD cells, we investigated the thalamic HD signal during periods of immobility and compared the spiking patterns of HD cells to the preceding exploration periods. Spindle oscillations (7–14 Hz), which appear during sleep stage 2 and during periods of immobility (high-voltage spindles) (Buzsaki 1991; Buzsaki et al. 1988), are generated by the thalamic reticular nucleus. They drive potent inhibitory postsynaptic potentials in thalamo-cortical neurons (Steriade et al. 1985). Here we explored whether, during high-voltage spindle periods, thalamic HD cells maintain their preferred firing direction and whether their firing patterns are triggered during spindle oscillations. Our data show that HD neurons are not entrained by the spindles, suggesting that thalamic HD signal is triggered by excitatory but not inhibitory inputs. On the basis of this finding, we exclude from our Hodgkin-Huxley model hyperpolarization-triggered low-threshold Ca^2+^ currents and instead include depolarization-triggered high-threshold Ca^2+^ currents as a factor that can affect spiking regularity. Our goal here is to test experimentally which would best predict the firing pattern of thalamic HD cells in behaving rats. Previously modeled ATI dynamics account for spike rate adaptation and the postinhibitory rebound of vestibular nucleus neurons (van der Meer et al. 2007). Here our model also relies on the role of spike rate adaptation; however, the triggering of the spike trains is linked not to inhibitory but to excitatory stimuli. Our findings suggest that the intrinsic calcium and adaptation currents, which evoke irregular firing, lead to higher firing rate at the ascending slope of a sinusoidal depolarization, inducing the separation angle of the HD cells. Our data show, for the first time, that the turn-specific modulation of the directional tuning curve depends on the degree of firing irregularity of HD cells together with their spike adaptation properties.

## MATERIALS AND METHODS

### 

#### Surgical implantation of electrodes.

**Fig. 1.**
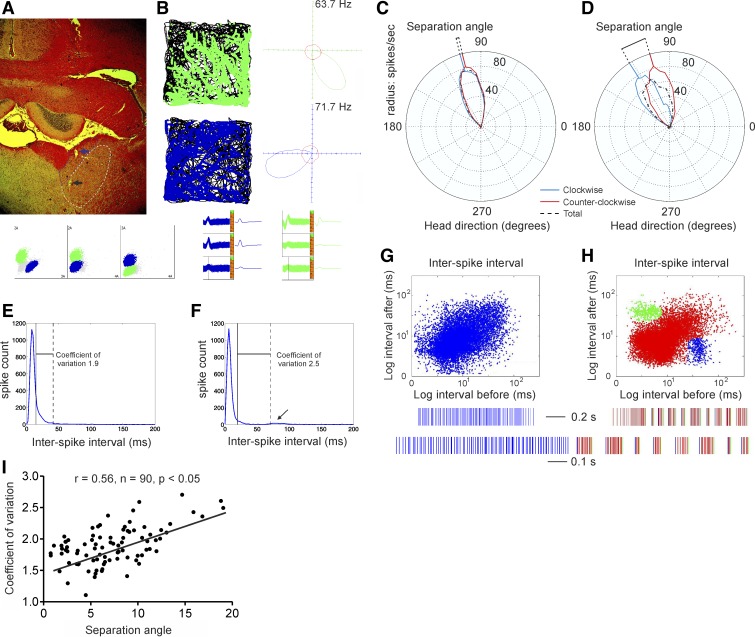
Turn-specific modulation of thalamic tuning curves correlates with spike variability. *A*: coronal brain section from a rat in which 8 chronically implanted tetrodes targeted the anterior thalamic nuclei. The electrode (blue arrow) was first positioned in anterodorsal nucleus (AD, dashed blue line) and then lowered (black arrow) to the medio-dorsal section of anteroventral nucleus (AV, dashed white line). *B*, *left*: the spiking of 2 cells (green and blue symbols) during experimental sessions conducted in a rectangular recording arena, where the animal's path is marked with a black line. *Right*: firing rate vs. head direction (HD) tuning plot of the same HD units. The polar plot for each cell represents the distribution of time heading in different directions across all time bins of the trial (red) and the distribution of HDs for time bins when a spike was recorded from the cell (blue and green). The coordinate system denotes maximal firing frequency of the recorded unit with 0 Hz in the center and 63.7/71.7 Hz in the periphery. *Bottom left*: sample scatterplot, showing signals recorded between 3 pairs of electrodes on a given tetrode. *Bottom right*: 2 sample waveforms of HD cells, corresponding to green and blue clusters in the scatterplot. *C* and *D*: sample polar plots of thalamic HD cells with a small (*C*) or large (*D*) separation angle. The tuning curve for clockwise (CW) turns is marked with blue and for counterclockwise (CCW) turns with red and the total with dashed black. The connecting black line represents the amplitude of the separation angle. *E*: histogram of interspike interval (ISI) for the cell from *C*. The vertical black line denotes mean ISI, while the dashed black line depicts the standard variation. The ratio between them is the coefficient of variation, visualized with the connecting horizontal line. *F*: histogram of ISI for the cell from *D*. The arrow indicates an ISI region, which is absent in *E*. *G*, *top*: 2-dimensional log-scale ISI scatterplot for the unit with a small separation angle. *x*-Axis indicates the interval to the previous action potential, and *y*-axis indicates the interval to the next one. *Bottom*: sample spike recordings of the same HD unit for 1-s duration (*top*) and 500-ms duration (*bottom*). *H*, *top*: ISI plots for the unit with a large separation angle. The main cluster is marked with red symbols, while the 2 additional clusters are marked in green and blue. *Bottom*: sample recordings of the same cell with 1-s duration (*top*) and 500-ms duration (*bottom*). Note that the spikes from the blue ISI cluster take first position in the spike trains, while the spikes from the green ISI cluster are positioned last. The intermediate spikes correspond to the points from the central red ISI cluster. *I*: positive correlation between the degrees of separation angle and the values of coefficient of variation for thalamic HD cells.

Experiments were conducted in accordance with European Community Directive 86/609/EC and the Cruelty to Animals Act, 1876 and were approved by the Bioresources Ethics Committee, Trinity College Dublin. Surgical implantation and single-unit recordings were performed as previously described (Tsanov et al. 2011b). The recording electrodes consisted of eight bundles of four platinum-iridium wires (90% platinum, 10% iridium; HM-L insulated, 25-μm bare wire diameter, California Fine Wire) twisted together. We used electrodes in a tetrode configuration, which allowed clear identification of thalamic units (Gray et al. 1995; McNaughton et al. 1983). Tetrodes were threaded through a 25-gauge guide cannula and protected with a 21-gauge cover. Tetrodes were then mounted in a small microdrive (Axona) and were implanted in AD (−1.5 AP, −1.3 ML, and 3.5 mm dorsoventral to dura) and AV (−1.5 AP, −1.4 ML, and 5.0 mm dorsoventral to dura) thalamus (Fig. 1*A*).

#### Recording techniques.

After at least 1 wk of recovery subjects were connected, via a 32-channel headstage (Axona), to a recording system that also allowed for animal position tracking. Signals were amplified (10,000–30,000×) and band-pass filtered between 380 Hz and 6 kHz for single-unit detection. To maximize cell separation, only waveforms of sufficient amplitude (at least 3 × noise threshold) were acquired (Fig. 1*B*). Candidate waveforms were discriminated off-line with graphical cluster-cutting software (Axona), which allows waveform separation based on multiple features including spike amplitude, spike duration, maximum and minimum spike voltages, and the time of occurrence of maximum and minimum spike voltages. Autocorrelation histograms (autocorrelograms), which represent the cross-correlation of a spike with itself, were calculated for each unit, and the unit was removed from further analysis if the histogram revealed the existence of correlations within the first 2 ms (refractory period), inconsistent with good unit isolation.

#### Recording sessions.

The recordings took place in a square arena (64 × 64 × 25 cm high) situated in the center of a room with multiple background cues and with an illumination level of 30–50 lx. Rats were placed in the open field, and 20-mg food pellets (TestDiet, Formula 5TUL) were thrown in every 20 s to random locations within the arena; in this way, animals locomoted regularly, allowing for complete sampling of the environment. To ensure proper sampling of the HD signal, we used only recordings where the animals locomoted at least 100 m per recording session. Two small infrared light-emitting diodes (LEDs), one brighter than the other, were attached to the rat's head for the purpose of tracking head position. The two LEDs were separated by 5–7 cm and identified by video camera and position-detection hardware (Axona) on the basis of their differential brightness. The position sampling rate was 50 Hz. Prior to the immobility sessions the administration of pellets was discontinued, and the animals were allowed to rest. Immobility and exploration sessions were conducted in the same recording arena, the animals allowed to rest in a prone position and to move between immobile periods. Each recording session was 16 min. During the immobile sessions the animals underwent several immobility periods with an average duration of 2–3 min per immobile session. Off-line analysis of local field potential (LFP) enabled detection spindle periods from the immobile sessions; the spiking properties of spindle periods were further analyzed. The identification of the spindle episodes was based on their amplitude (>100 μV), frequency (≥5 and ≤20 Hz), and duration (≥1 s). Thus immobile sessions with duration of 16 min contain several spindle periods, each of them with different HDs due to the animal's head movement between the immobile periods. The animals' heading during each immobile period appeared random, and therefore the probability of recording every HD unit during its preferred direction was also random. We analyzed the spindle periods if at least one of them was in the preferred firing direction of the recorded HD unit. The LFP recordings were performed as previously described (Tsanov et al. 2011b). Information was displayed as the magnitude of the short-time Fourier transform in a color gradient graph with the maximum corresponding to 0 dB and the minimum to −60 dB.

#### Head direction analyses.

Directional analyses were performed for all HD cells in AD and AV thalamic nuclei, 90 units in total. The rat's HD was calculated for each tracker sample from the projection of the relative position of the two LEDs onto the horizontal plane (Fig. 1*B*). The directional tuning function for each cell was obtained by plotting the firing rate as a function of the rat's directional heading, divided into bins of 5° (Fig. 1, *C* and *D*). The firing rate was computed as the total number of spikes divided by the total time in that bin (Taube et al. 1990). To restrict the influence of inhomogeneous sampling on directional tuning, we accepted data only if all directional bins were sampled by the rat. The directionality of the head turns was defined with a polar coordinate system, such that increasing angles indicate CCW turns and decreasing angles indicate CW turns (Fig. 1*C* and *D*). The background firing rate was calculated as <10% from the peak firing rate. The momentary angular velocity of the animal's head was calculated as the difference in the angle of HD between successive 50-Hz time samples. The direction time series was first smoothed by calculating a five-point running average. After smoothing, the instantaneous velocity was calculated as the angular displacement between two successive points per time (Taube 1995). Positive angular velocities are for CCW turns, and negative angular velocities are for CW turns. The separation angle was calculated as the difference between a cell's CW mean direction and its CCW mean direction (Blair and Sharp 1995). We used a Gaussian mean method where the CW and CCW firing rate vs. HD functions were treated as Gaussian distributions. The mean of each distribution was calculated from all directional bins, and the difference between the two means gave an estimate of the separation angle (Taube and Muller 1998).

#### Head direction normalization.

The data from exploration recording sessions were plotted in a normalized directional tuning curve of firing rate vs. HD tuning for all HD cells. The normalized firing rate is the firing rate divided by the maximal firing rate for each unit. The HD in degrees (of cells that respond to different heading directions) was aligned with the peak of the tuning curve for all units to a HD of 180° (Bassett et al. 2005). The data from immobile sessions included only off-line filtered spindle periods. The firing rate of each cell for the immobile sessions was normalized (transformed into %), considering the peak firing rate from the preceding exploration recording session as 100%. Similarly, the preferred firing direction of each cell for the immobile sessions was aligned to 180°, using the same shift in degrees that aligns the preferred firing direction from the preceding exploration recording session to 180°. The arbitrary separation angle value of 8° that differentiated low- and high-value groups was based on the rounding of the median value (7.6°) of the Poisson distribution for all separation angles from the recorded thalamic units.

#### Interspike interval analysis.

ISIs were calculated as the time interval of the spike to the preceding and to the following spike. The variability of ISIs was quantified with the ratio of the standard deviation to the mean of ISIs (coefficient of variation). To avoid the distorting effect of very large ISIs from the background firing rate on the mean coefficient of variation we used two strategies: *1*) we included all the ISIs from the series of spikes that fell within the range of the HD tuning curve for a given unit (directional firing range). The directional firing range was defined as the range with a firing frequency ≥10% of the peak firing frequency from the unit's preferred HD. Based on the individual HD range, the coefficient of variation calculation included data from the directional firing range, excluding the Gaussian tails, characterized by background firing. ISIs longer than 1,000 ms, inconsistent with the spiking patterns within HD range, were filtered out. *2*) For the second coefficient of variation analysis, we included all the ISIs from the entire recording for a given unit after filtering the ISIs for the range of <500 ms. In this way the coefficient of variation calculation did not include ISIs longer than 500 ms, which is a common feature of background firing rate. The coefficient of variation analysis for ISIs indexes the degree of irregularity, with higher coefficient variation values indicating a greater degree of irregular firing (Shadlen and Newsome 1994; Softky and Koch 1993).

#### Computational model.

The computational model was run under the NEURON simulation environment (Hines and Carnevale 1997) (see appendix). The model described here is a single-compartment neuron (cylinder of diameter *d* and length *L*) described by the following membrane equation (Pospischil et al. 2008):

$$C_{\text{m}}\left(\text{d}V/\text{d}t\right)=-g_{\text{leak}}\left(V-E_{\text{leak}}\right)-I_{\text{Na}}-I_{\text{Kd}}-I_{\text{M}}-I_{\text{L}}$$

where *V* is the membrane potential, *C*_m_ = 1 mF/cm^2^ is the specific capacitance of the membrane, *g*_leak_ is the resting (leak) membrane conductance, and *E*_leak_ is its reversal potential. The model considered here is a reduced type of biophysical model where the intrinsic properties arise from voltage-dependent conductances, each described by differential equations (Hodgkin-Huxley-type models). We use a one-compartment Hodgkin-Huxley- type model, which precisely represents the biological dynamics of main neuronal currents and at the same time simplifies the functional subdivision of the modeled neurons to regular or irregular behavior (Pospischil et al. 2008). *I*_Na_ and *I*_Kd_ are the sodium and potassium currents responsible for action potentials, *I*_M_ is a slow voltage-dependent potassium current responsible for spike-frequency adaptation, and *I*_L_ is a high-threshold, L-type calcium current. These voltage-dependent currents are variants of the same generic equation:

$$I_{j}=g_{j}m^{M}h^{N}\left(V-E_{j}\right)$$

where the current *I*_*j*_ is expressed as the product of, respectively, the maximal conductance *g*_*j*_, activation (*m*) and inactivation variables (*h*), and the difference between the membrane potential *V* and the reversal potential *E*_*j*_. The gating of the channel is derived from the following first-order kinetic scheme: *C*α(*V*) ↔ β(*V*)*O*, where *O* and *C* are the open and closed states of the gate. The variables *m* and *h* represent the fraction of independent gates in the open state (Hodgkin and Huxley 1952). The steady-state activation and the time constant are, respectively, given by *m*∞ = α/(α + β) and τ*m* = 1/(α + β), and similarly for *h*. Additional details of the computational model are described in the appendix.

#### Statistical analyses.

Statistical significance was estimated by two-tailed Student's *t*-test and two-way analysis of variance (ANOVA), paired with post hoc Newman-Keuls test. The probability level interpreted as significant was *P* < 0.05. Data points are plotted ± SE.

## RESULTS

### 

#### Turn-specific modulation of head direction tuning curve.

A total of 90 well-isolated HD units were recorded from AD (48 units) and AV (42 units) thalamic nuclei in 12 rats (Fig. 1, *A* and *B*). We compared the difference in the tuning curve of thalamic neurons between CW and CCW turns, known as the separation angle (Blair and Sharp 1995; Taube and Muller 1998). The average separation angle of all recorded HD cells is 7.12 ± 0.4°. The histological verification suggests that the AV nucleus is characterized by units with a larger separation angle (8.67 ± 0.65°) than the AD nucleus (5.96 ± 0.51°). Our aim is to understand why some HD units express small differences in their preferred firing direction between CW and CCW turns (Fig. 1*C*) while others express larger turn-specific differences (Fig. 1*D*).

The variability of ISIs of thalamic HD cells was quantified with the coefficient of variation (the ratio of the standard deviation to the mean of ISIs; see materials and methods). Previous analysis of ISI variability of HD spiking was restricted to the peak of the tuning curves with a range of ±6° from the preferred firing direction (Taube 2010). By including the ISIs from the tuning curve with >10% of the peak firing rate in the coefficient of variation, we investigated here the contribution of the firing patterns in the tuning curve's ascending and descending slopes to the formation of separation angle in the HD cells. The coefficient of variation for HD cells with small separation angles (Fig. 1*E*) is smaller compared with HD cells with larger separation angles (Fig. 1*F*). While the coefficient of variation indicates the higher variability of ISIs between the two groups, the ISI scatterplots show the interspike ranges that increase the standard deviation of the ISIs' mean value (see arrow in Fig. 1*F*). Two-dimensional ISI scatterplots of HD cells with lower variability are characterized by one major ISI cluster (Fig. 1*G*), while ISI scatterplots of HD cells with higher variability express two more ISI clusters (Fig. 1*H*, *top*). The additional clusters represent the first/last spike of the rhythmic spike trains in the theta range (5–10 Hz; Fig. 1*H*, *bottom*), which are a characteristic feature of HD-by-theta cells (Tsanov et al. 2011a). Although ISI scatterplots visualize the rhythmicity-related clusters, the coefficient of variation detects the differences of spiking irregularity between recorded neurons. We next correlated the value of the coefficient of variation, calculated for the ISIs within the directional firing range, to the separation angle for all recorded HD units (Fig. 1*I*) and found a significant positive correlation (Pearson, *r* = 0.567, *n* = 90, *P* < 0.001). The values of the coefficient of variation range mainly between 1.5 and 2.5, suggesting a large standard deviation of the mean firing rate. To reduce the effect of ISI outliers on the mean firing rate, we used a parallel approach in estimating the ISIs for the variance analysis. For this we used ISIs with length of <500 ms, where the ISIs longer than 500 ms were filtered out. The application of this filter reduced the coefficient of variation values to the range between 1 and 2. Importantly, the correlation between the coefficient of variation and the separation angle was well preserved (Pearson, *r* = 0.560, *n* = 90, *P* < 0.001), suggesting that the result is not biased by long ISI outliers. Location-specific analysis shows that AV neurons express a larger coefficient of variation (1.93 ± 0.04 for directional filter and 1.73 ± 0.04 for ISI filter) compared with AD neurons (1.84 ± 0.04 for directional filter and 1.64 ± 0.05 for ISI filter).

This finding suggests that ISI variability (which reflects the irregular firing of the neurons) is a factor that relates closely to the formation of the separation angle. ISI variability may reflect the synergistic formation of groups of spikes and the intervals between these groups. Thus we evaluated subsequently the mechanisms that generate the formation of irregular firing patterns (see materials and methods).

#### Angular velocity effect on thalamic directional firing frequency in behaving rats.

**Fig. 2.**
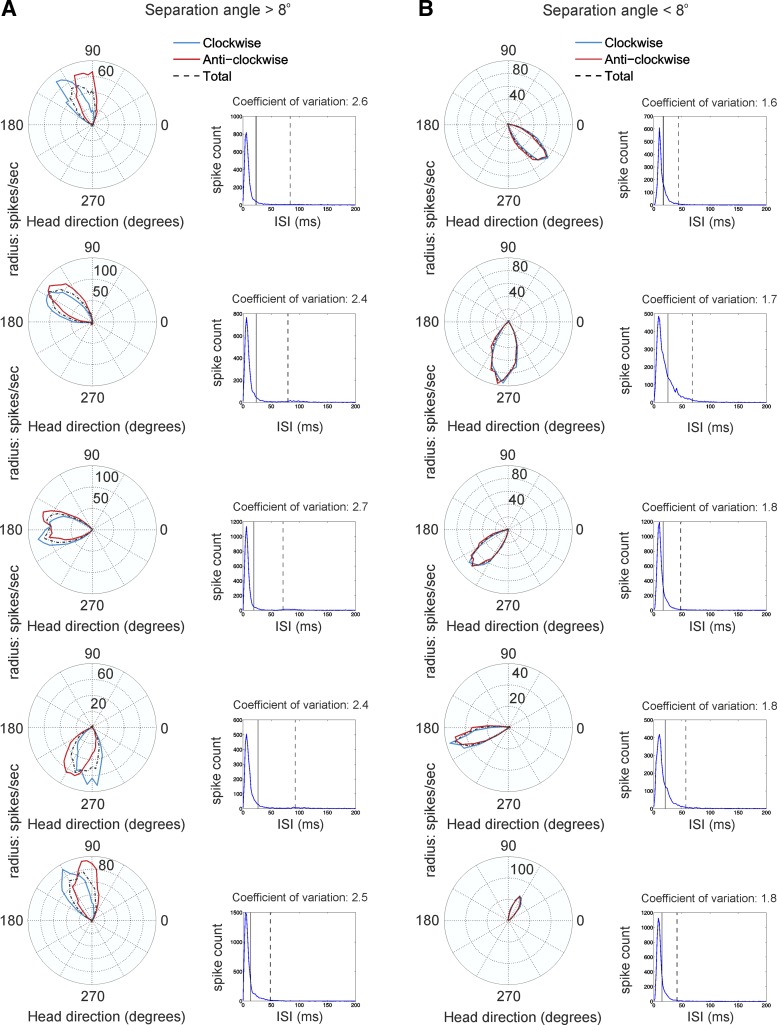
Separation angle and ISI variability in anterior thalamus. *A* and *B*: sample polar plots of 5 thalamic HD cells with a separation angle > 8° (*A*), represented also as the top 5 points with the highest separation angle in Fig. 1*I*, and 5 HD cells with a separation angle < 8° (*B*), represented also as the bottom 5 points with the lowest separation angle in Fig. 1*I*. The tuning curve of CW turns is marked with blue, the CCW turns in red, and the total in dashed black. *Left*: polar plots. *Right*: ISI histograms of the same cells with a separation angle > 8° (*A*) and with a separation angle < 8° (*B*). The vertical black line denotes the mean ISI, while the dashed black line shows the standard variation.

**Fig. 3.**
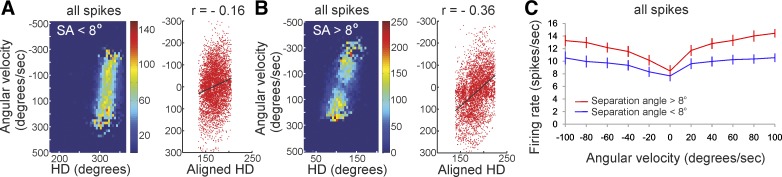
Angular velocity effect on HD cells depends on the separation angle. *A*: color-coded scatterplot of spiking frequency plotted against head direction (*x*-axis) and angular velocity (*y*-axis) from a sample unit with a separation angle (SA) < 8°. Scatterplot on *right* represents the correlation between the angular velocity and the aligned head direction (centered to 180°) for the recorded spikes (red symbols). *B*: color-coded scatterplot of spiking frequency (*left*) and for aligned head direction (*right*) from a sample unit with a separation angle > 8°. The Pearson's correlation between angular velocity and head direction is shown. *C*: average firing frequency vs. angular velocity plot of all units with a separation angle < 8° (*n* = 56) against all units with a separation angle > 8° (*n* = 34) (means ± SE).

Thalamic HD signal is linked to changes of angular head velocity (Taube 1995), and there is a linear relationship between separation angle and angular velocity for AD thalamic cells (Blair and Sharp 1995). Thus we also investigated here the degree of modulation of the HD tuning curve in relation to angular velocity. We next divided the HD units into two groups, using a separation angle of 8° as a distinguishing value (see materials and methods). The presumption for such an approach is to evaluate the effect of angular velocity on the firing frequency in relation to the separation angle between the groups of units with large vs. small separation angle. Concordantly, the group of units with separation angle > 8° (Fig. 2*A*) is characterized with higher values of coefficient of variation (Fig. 2*A*, *right*), while the units with separation angle < 8° (Fig. 2*B*) express a lower coefficient of variation (Fig. 2*B*, *right*). We compared the correlation of angular velocity and spiking parameters between the group of units with separation angle < 8° (Fig. 3*A*) and the group of units with separation angle > 8° (Fig. 3*B*). By shifting the tuning curves, we can analyze the angular velocity for several neurons with different preferred HDs for both groups. For this purpose, we aligned all tuning curves to center their peaks at 180° (Fig. 3, *A* and *B*, *right*). The Pearson correlation between angular velocity and normalized HD of all spikes for the group of units with separation angle > 8° is *r* = −0.258 (*n* = 314,479), while the correlation for the separation angle < 8° group is *r* = −0.140 (*n* = 501,218). To test the null hypothesis that there is no significant difference between both correlations, we converted the *r* values into *Z* scores (*Z* = −0.264 for the group of units with separation angle > 8° and *Z* = −0.141 for the group of units with separation angle < 8°). We calculated the difference between both correlations over the standard error (see materials and methods) and found the *Z*_|difference|_ = 53.91, which is significant (*t*-test, *P* < 0.001) at the 99% confidence level, thus rejecting H_0_. The difference in relation to angular velocity between both groups can be visualized by the average frequency in degrees per second (Fig. 3*C*), and the statistical significance is represented by repeated-measures ANOVA [*F*_(1,10)_ = 12.75, *P* < 0.01, *n* = 56/34]. These data demonstrate that the firing rate of neurons with more irregular firing patterns is better correlated with angular velocity compared with the firing rate of neurons with less irregular firing.

We also correlated the steepness of the angular velocity to the coefficient of variation values for all HD cells. The steepness was expressed as the slope of the function between the firing frequency and angular velocity (Bassett and Taube 2001). We found a significant, positive correlation (Pearson, *r* = 0.445, *n* = 90, *P* < 0.001). This finding suggests that the angular velocity and the separation angle are both related to the interspike patterns of the HD cells.

#### Directional firing of thalamic head direction cells during spindle periods.

**Fig. 4.**
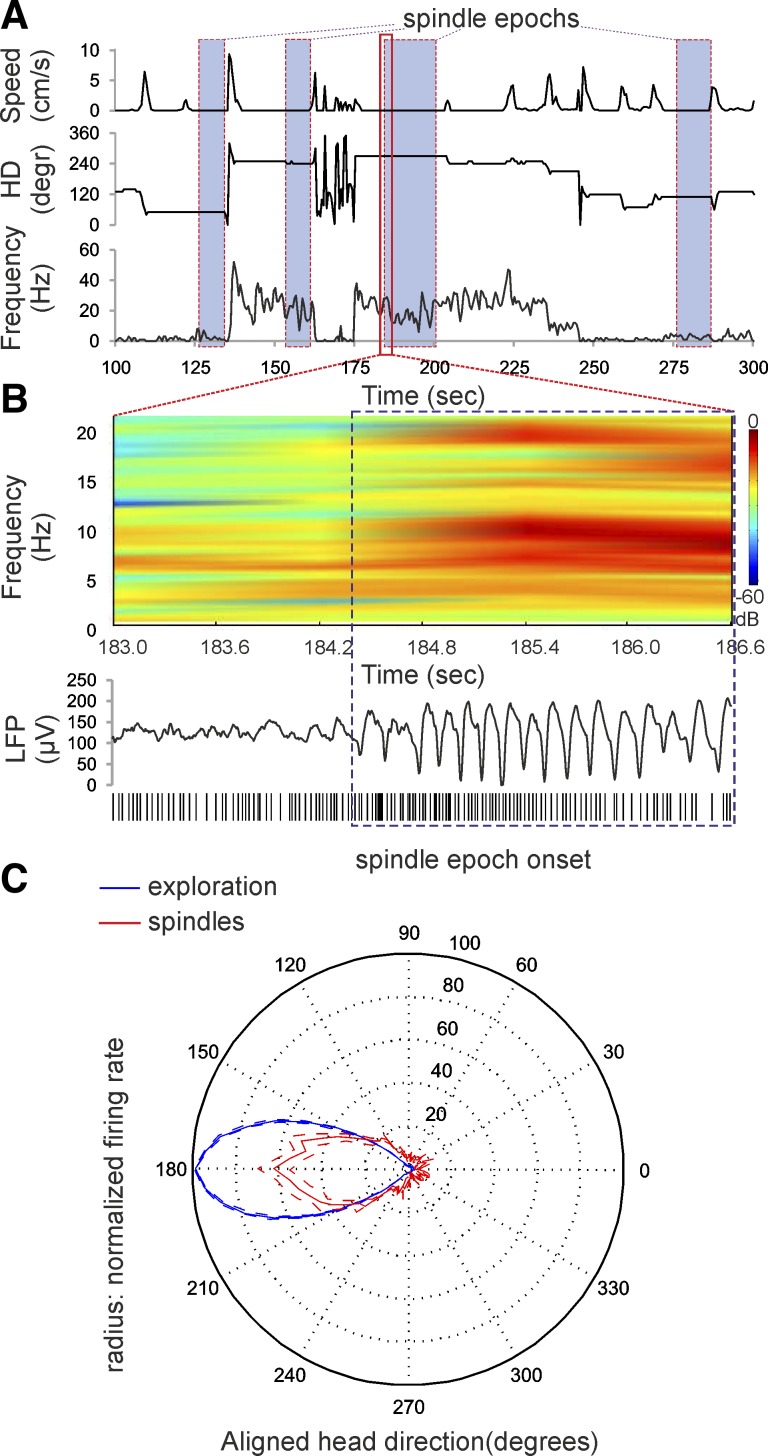
Thalamic HD signal is preserved during spindle periods. *A*: sample recording of speed (*top*), HD (*middle*), and HD unit firing frequency (*bottom*) during immobility period. The occurrence of spindle epochs is denoted by blue-filled rectangles. The beginning of the third spindle epoch is marked by the red rectangle and magnified in *B*. *B*, *top*: the onset of a spindle period is represented by a sample color-coded power spectrogram recording of local field potential (LFP) in the AD thalamic nucleus. *B*, *bottom*: LFP trace shows the oscillatory profile of spindle onset, corresponding to the power spectrogram above. The spikes of the simultaneously recorded HD unit are plotted below the LFP trace. The blue dashed rectangles indicate the identification of the spindle period. *C*: firing rate vs. HD polar plot of all HD cells for exploration periods and spindle periods. The HD is aligned with the peak of the tuning curve for all units set to 180° (means ± SE).

To test the hypothesis that thalamic HD irregular firing is induced by hyperpolarizing inputs, we investigated HD spiking patterns during spindle periods. Inhibition in the anterior thalamus depends predominantly on extrinsic circuits, the most potent of which arise from the thalamic reticular nucleus, and the periods of most robust reticular activity are associated with thalamo-cortical spindles. They are a characteristic feature of thalamic oscillations during immobility periods and during stage 2 of slow-wave sleep. While we compared the thalamic activity between spindle periods and the preceding exploration periods, we did not intend to compare the firing properties between sleep and awake states. After the pellet chasing (exploration) sessions the administration of pellets was discontinued, and the animals were allowed to rest in the same recording arena. The immobility sessions were characterized by brief episodes in which the animals were immobile in a prone position and several brief mobile periods. The definition of high-voltage spindle periods (Fig. 4*A*) in our study was based on off-line detection of the characteristic LFP (see materials and methods) (Fig. 4*B*). The peak of the spindle spectral power is in the range of 7–14 Hz, with a gradual decrease of spindle frequency after the onset of spindle epochs (Fig. 4*B*, *top*). The spindle power spectrogram shows a parallel spectral power increase in the range of 12–22 Hz (Fig. 4*B*, *top*), which is a typical feature of thalamic spindles as harmonic oscillations (Tsanov et al. 2011b). For the directional firing range the HD units continued to fire with similar rate during the transition from exploration periods to immobile spindle periods (Fig. 4*B*, *bottom*), as well as throughout the entire immobile episodes. During subsequent immobile periods, the animal's head was positioned in different directions across the horizontal plane, including the preferred firing direction for the analyzed unit. However, the sampling number of the HD degrees is low (few HDs with spindle oscillations), which is insufficient to evaluate the changes of the firing rate between the exploration and the spindle episodes. Therefore, we normalized the firing rate and head directionality for all cells from all animals (see materials and methods), aligning the peak firing rate for all units to a HD of 180° (Bassett et al. 2005). The integration of all spindle periods for all rats in an average tuning curve showed a significant decrease of the firing rate for the preferred direction [ANOVA, *F*_(1,72)_ = 20.03, *P* < 0.001, *n* = 64] compared with the averaged tuning curve for all exploration periods (Fig. 4*C*). The average peak firing rate during spindles (62.5 ± 7.3%) was ∼40% lower than the average peak firing rate for exploration periods. The decrease of the firing rate was significant in the range from 160 to 200 normalized degrees (Newman-Keuls test, *P* < 0.01).

**Fig. 5.**
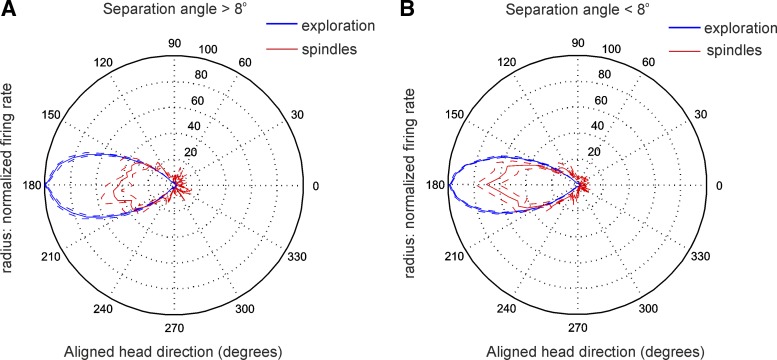
Spindle modulation of directional tuning curve for thalamic HD cells. *A* and *B*: firing rate vs. HD polar plot of all cells with separation angle > 8° (*A*) and of all cells with separation angle < 8° (*B*) for exploration periods and spindle periods. The HD is aligned with the peak of the tuning curve for all units set to 180° (means ± SE).

Analysis of the normalized tuning curve for HD cells with a separation angle > 8° revealed a reduced firing rate (46 ± 10.5%; Fig. 5*A*) compared with HD cells with a separation angle < 8° (70 ± 8.9%; Fig. 5*B*). The reduced firing frequency of HD cells during spindle epochs suggests that hyperpolarization-mediated mechanisms may not be involved in HD spike generation. To test this hypothesis, we next analyzed the relationship of HD spiking to the phase of spindle oscillation.

#### Spiking properties of anterodorsal thalamic units in relation to the spindle cycle.

**Fig. 6.**
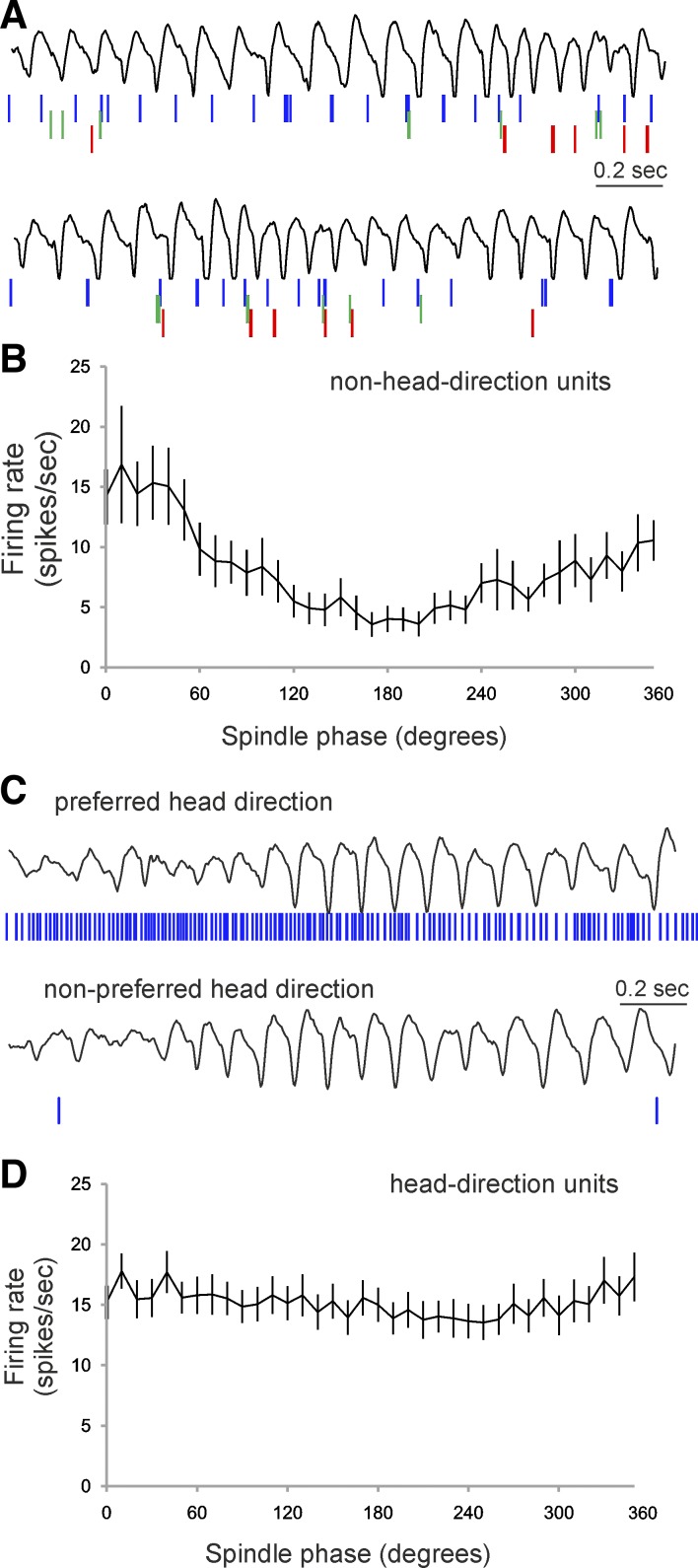
Thalamic HD cells are not phase-locked to spindle oscillations. *A*: examples of LFP traces (*top*) and the parallel spiking activity (*bottom*) of non-HD units. The colors indicate the spikes of different units. The lower LFP spikes example represents the same recording during different heading direction. *B*: average relationship between spindle phase and the firing rate of thalamic non-HD units, where the peaks of 1 spindle cycle are indicated as 0° and 360°, with the trough as 180°. *C*: sample examples of LFP traces (*top*) and parallel spiking activity (*bottom*) of a HD unit. The upper LFP/spikes example represents recording during the preferred firing direction, while the lower example shows recording during the nonpreferred firing direction. *D*: average relationship between spindle phase and the firing rate of thalamic HD units.

We determined the position of each spike relative to the spindle cycle (0° and 360° indicated the peaks, while 180° indicated the trough of 1 oscillatory cycle) (Harris et al. 2002). Raw spiking traces (Fig. 6*A*) and their phase analysis (Fig. 6*B*) revealed that non-HD cells in the anterior thalamus are phase-locked to the spindle oscillations, with a preference for the positive phase. The firing rate for this group is higher for the positive (270–90°) compared with negative (90–270°) phases of the spindle oscillations [ANOVA, *F*_(1,36)_ = 12.75, *P* < 0.01, *n* = 12]. The HD units spike continuously during the spindle episodes when the animal's heading is in the unit's preferred firing direction (Fig. 6*C*, *top*). The spiking of HD units is not driven by the spindle rhythm in the unit's nonpreferred firing direction, where the firing rate is very low (Fig. 6*C*, *bottom*). We analyzed the relationship between HD spikes and spindle oscillations (Fig. 6*D*) and found no significant difference in firing rates for the positive (270-90°) compared with negative (90–270°) phases (ANOVA, *F* < 1, *P* > 0.05, *n* = 37). We also found no significant correlation between the spindle amplitude and the firing rate (Pearson, *r*^2^ = 0.015, *P* < 0.05, *n* = 37). These results do not provide evidence for HD spiking in phase with spindle oscillations during spindle epochs, suggesting that the firing of HD cells might be unrelated to reticular nucleus-mediated inhibition.

#### Simulation of HD tuning curve in a single-compartment Hodgkin-Huxley model.

While the inhibitory input during high-voltage spindles does not potentiate the firing of thalamic neurons, the intrinsic low-threshold calcium current is an unlikely scenario for the irregularity of thalamic spikes. Here we model the alternative mechanism that is mediated by excitatory input, proposing that the spiking irregularity results from high-threshold calcium and slow potassium currents. We therefore included depolarization-triggered high-threshold Ca^2+^ current in our single-compartment model (Pospischil et al. 2008). We modeled an irregular firing neuron (see materials and methods) exhibiting *1*) Na^+^ and K^+^ currents for generating spikes, *I*_Na_ and *I*_Kd_, *2*) slow K^+^ current activated by depolarization, responsible for spike-frequency adaptation, *I*_M_, and *3*) high-threshold Ca^2+^ current, *I*_L_.

**Fig. 7.**
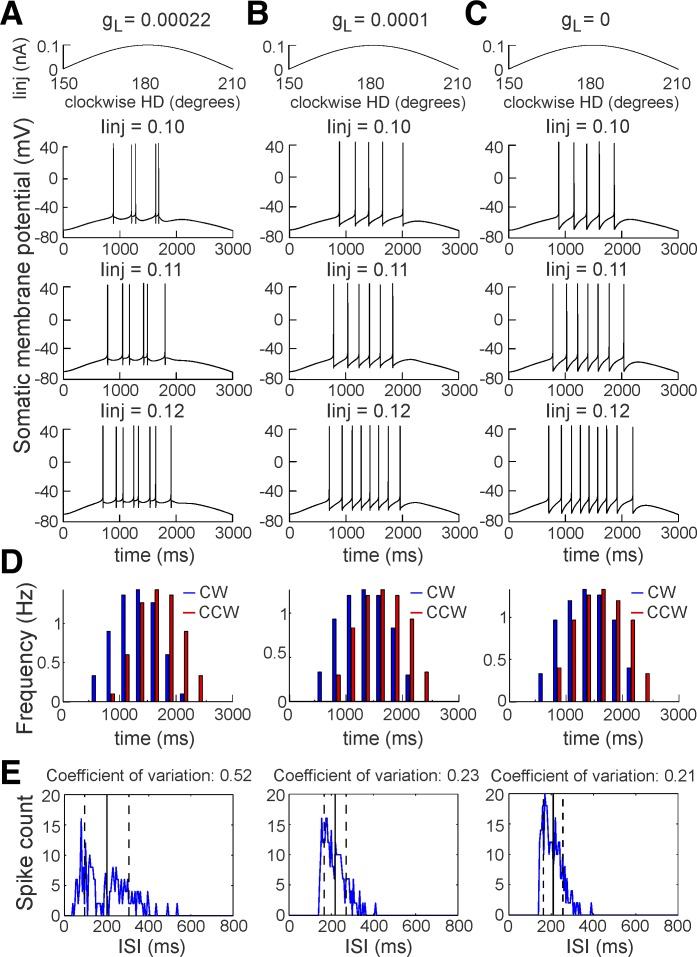
Model of irregular spiking HD cell based on thalamic anterior thalamus. *A*: sinusoidal current injection (*I*_inj_) to a neuron with a high-threshold calcium channel conductance of *g*_L_ = 0.00022 S/cm^2^. *Top* trace represents the current application where the start is considered at 150° for CW simulations and 210° for CCW simulations. *Bottom* panels represent the evoked spiking with different *I*_inj_, with values of 0.10 nA (*top*), 0.11 nA (*middle*), and 0.12 nA (*bottom*). *B*: sinusoidal current injection to the neuron with *g*_L_ = 0.0001. *C*: sinusoidal current injection to the neuron with *g*_L_ = 0. *D*: histograms of averaged firing frequency for 30 simulations with small *I*_inj_ variations (0.001-nA step) for CW and CCW simulations for *g*_L_ = 0.00022 S/cm^2^ (*left*), *g*_L_ = 0.0001 S/cm^2^ (*center*), and *g*_L_ = 0 S/cm^2^ (*right*). *E*: ISI histograms of averaged firing frequency for 30 simulations with small *I*_inj_ variations (0.001-nA step) for *g*_L_ = 0.00022 S/cm^2^ (*left*), *g*_L_ = 0.0001 S/cm^2^ (*center*), and *g*_L_ = 0 S/cm^2^ (*right*). The vertical solid black line denotes the mean ISI, while the dashed black lines represent the standard variation.

To simulate the biological input pattern of the HD cells, we applied injection current (*I*_inj_) with a sinusoidal shape, mimicking the gradual increase and subsequent gradual decrease of the depolarizing inputs during the cell's preferred HD in the horizontal plane. We set the duration of *I*_inj_ at 3,000 ms and applied current amplitude with small variations, similar to the biological conditions in which the depolarizing inputs express natural variation. In total, we applied 30 simulations with amplitude change from 0.092 to 0.123 nA in steps of 0.001 nA. The peak of the sinusoidal current is analogous to normalized HD, where the peak of the tuning curve responds to 180°. The start of *I*_inj_ application corresponds to 150° and the end to 210° for CW modeling (Fig. 7, *A–C*, *top*). The CCW *I*_inj_ application is in reverse order: the start corresponds to 210° and the end to 150°. For our model, we generated irregular firing type behavior using *I*_L_ with high (*g*_L_ = 0.00022 S/cm^2^) and moderate (*g*_L_ = 0.0001 S/cm^2^) conductance. We also generated regular firing behavior by deactivation of *I*_L_ (*g*_L_ = 0 S/cm^2^). The application of *I*_inj_ induced spike doublets for high *g*_L_ (Fig. 7*A*), particularly at the peak of the injection sinusoidal current. Moderate conductance *g*_L_ was characterized by the absence of irregular ISI patterns (Fig. 7*B*), which was the case with regular firing cells (Fig. 7*C*). Our simulations showed that spikes within bursts (spike doublets) occurred preferentially on the ascending slopes of *I*_inj_, whereas regular spikes showed little slope selectivity. The concurrent activity of *I*_L_ and *I*_M_ gradually reduces the formation of irregular spike trains, reducing the spike count on the descending slope of the sinusoidal input. Such phase lead is evident in the average spike count histogram for all 30 simulations from the high-*g*_L_ group (Fig. 7*D*, *left*) and is less strongly expressed in the moderate-*g*_L_ and zero-*g*_L_ groups (Fig. 7*D*, *center* and *right*, respectively). Therefore, by regulating the depolarization of membrane potential, the degree of *g*_L_ helps determine the degree of ISI variation (Fig. 7*E*). High *g*_L_ results in a higher coefficient of variation (Fig. 7*E*, *left*) compared with the moderate-*g*_L_ ISI coefficient of variation (Fig. 7*E*, *center*), while zero *g*_L_ leads to the lowest value of ISI coefficient of variation (Fig. 7*E*, *right*).

#### Separation angle in modeled neurons depends on concurrent activity of high-threshold calcium and spike adaptation currents.

**Fig. 8.**
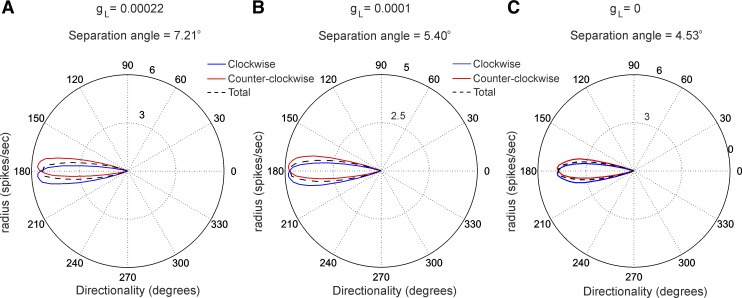
Separation angle depends on the high-threshold calcium channel conductance: polar plots of averaged firing frequency for 30 simulations with small *I*_inj_ variations (in 0.001-nA steps) for CW and CCW simulations for *g*_L_ = 0.00022 S/cm^2^ (*A*), *g*_L_ = 0.0001 S/cm^2^ (*B*), and *g*_L_ = 0 S/cm^2^ (*C*). The separation angles are 7.21°, 5.40°, and 4.53°, respectively.

**Fig. 9.**
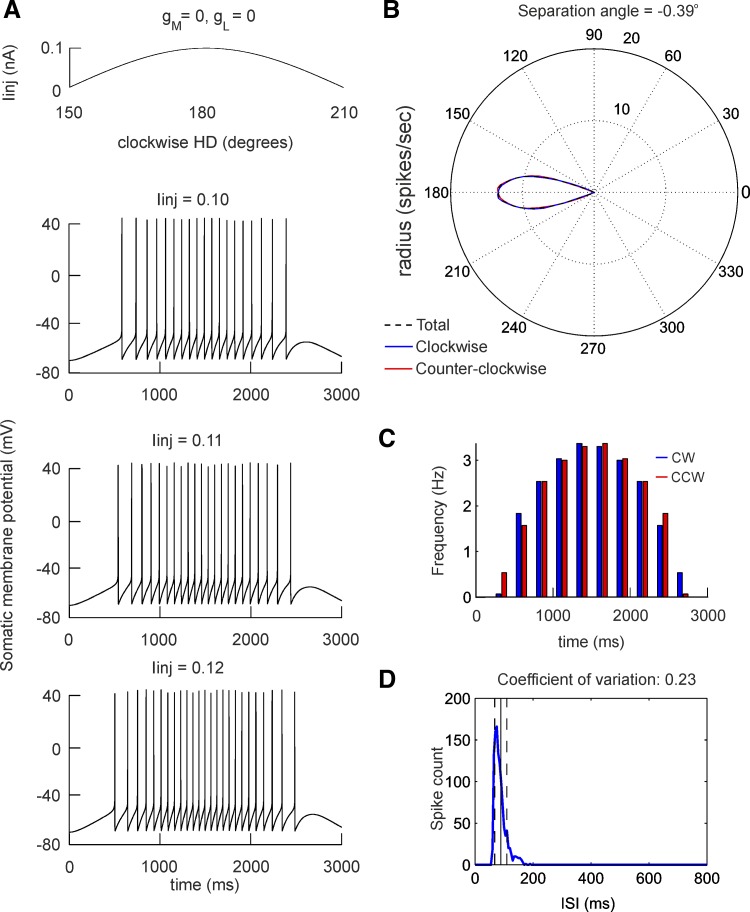
Directional properties of modeled regularly firing neuron. *A*: sinusoidal current injection (*I*_inj_) to neuron with high-threshold Ca^2+^ channel conductance *g*_L_ = 0 and slow K^+^ adaptation channel conductance *g*_M_ = 0. *Top* trace represents the current application where the start is considered at 150° for CW simulation and 210° for CCW simulation. *Bottom* panels represent the evoked spiking with different *I*_inj_ with values of 0.10 nA (*top*), 0.11 nA (*middle*), and 0.12 nA (*bottom*). *B*: polar plot of averaged firing frequency for CW and CCW simulations. The separation angle is −0.39°. *C*: histogram of averaged firing frequency for 30 simulations with small *I*_inj_ variations (0.001-nA step) for CW and CCW simulations for *g*_L_ = 0 and *g*_M_ = 0. *D*: ISI histogram for this neuron. The vertical solid black line denotes the mean ISI, while the dashed black lines denote the standard variation.

We next measured the phase lead for CW and CCW simulations in the same manner as we evaluate the turn-specific peak separation of the HD tuning curve (Fig. 8). For the high-*g*_L_ group separation angle was 7.21° (Fig. 8*A*), for the moderate-*g*_L_ group 5.40° (Fig. 8*B*), and for the zero-*g*_L_ group 4.53° (Fig. 8*C*), revealing a significant positive correlation (Pearson, *r* = 0.97, *n* = 90, *P* < 0.001). These data support experimental evidence that the formation of separation angles is dependent on the generation of Ca^2+^-dependent depolarization irregular spike trains. The fact that even the group with zero *g*_L_ value expresses separation angle suggests that another fundamental current is involved in this process. The role of adaptation current has been already modeled in both head-directional anticipation (van der Meer et al. 2007) and firing phase lead to sinusoidal inputs (Kepecs et al. 2002). To test the role of slow K^+^ current, responsible for spike-frequency adaptation, *I*_M_ in the generation of phase lead to the sinusoidal current injection we inactivated *I*_M_ in our model. The outcome was a separation angle close to 0 for the regularly firing cell (Fig. 9, *A* and *B*), with fully lost phase lead when both *I*_M_ and *I*_L_ are inactivated (Fig. 9*C*). Importantly, this result was paralleled by a low value of the ISI coefficient of variation (Fig. 9*D*), confirming the relation between interspike patterns and the turn-specific modulation of the modeled neurons.

#### Angular velocity effect on thalamic directional firing frequency in modeled neurons.

**Fig. 10.**
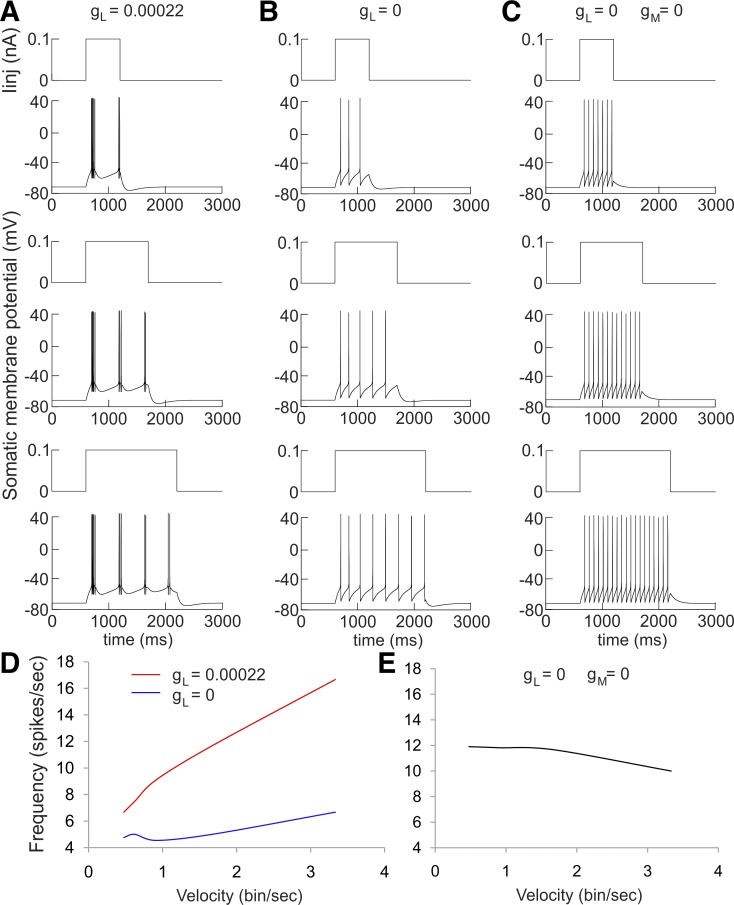
Velocity effect in modeled neurons. Continuous current injection to neuron with high-threshold Ca^2+^ channel conductance of *g*_L_ = 0.00022 S/cm^2^ (*A*), *g*_L_ = 0 S/cm^2^ (*B*) and *g*_L_ = 0 S/cm^2^ and *g*_M_ = 0 S/cm^2^ (*C*) for duration of 600 ms (*top*), 1,200 ms (*middle*), and 1,800 ms (*bottom*). *D*: firing frequency vs. velocity (bin/s) plot for *g*_L_ = 0.00022 S/cm^2^ and *g*_L_ = 0 S/cm^2^. *E*: firing frequency vs. velocity (bin/s) plot for *g*_L_ = 0 S/cm^2^ and *g*_M_ = 0 S/cm^2^.

We next used a modeled neuron to explain how the spiking of thalamic HD cells in relation to angular velocity depends on the irregular patterns of the spike trains. For this purpose, we simulate spiking in the preferred HD in a bin per second (similarly to the experimental analysis where the measurement is degree per second). A short duration of 600 ms in this bin leads to restricted spiking (Fig. 10*A*, *top*); however, twice the duration (1,200 ms) does not result in doubled spike counts (Fig. 10*A*, *middle*). Furthermore, a longer duration of 1,800 ms revealed a reduction in the number of spikes in the rhythmic spike trains with time (Fig. 10*A*, *bottom*). Therefore, continuous within-bin spiking adaptation, corresponding to the preferred HD, will lead to lower spiking frequencies (as is the case with HD firing during sleep periods). The potent role of the high-threshold Ca^2+^ current, *I*_L_, in establishing velocity-dependent firing frequency is shown when *I*_L_ is inactivated, i.e., *g*_L_ = 0 (Fig. 10*B*). In this case, the regular firing undergoes weak adaptation, which can be detected for longer durations (Fig. 10*B*, *bottom*). Thus the positive linear relationship between neuronal spiking and velocity (bin/s) is higher in irregular neurons expressing *I*_L_ compared with regularly firing neurons (*t*-test, *P* < 0.001; Fig. 10*D*). Finally, without spike-adaptation current conductance (*g*_M_ = 0) the firing frequency does not depend on the duration of the injected current (Fig. 10*C*) and the positive correlation between neuronal spiking and velocity is fully lost when both *I*_L_ and *I*_M_ are inactivated (Fig. 10*E*). In conclusion, our data show that the high-threshold calcium and adaptation currents together increase the correlation between the firing rate and the angular velocity.

#### Continuous sinusoidal but not phasic injection current induces phase lead.

**Fig. 11.**
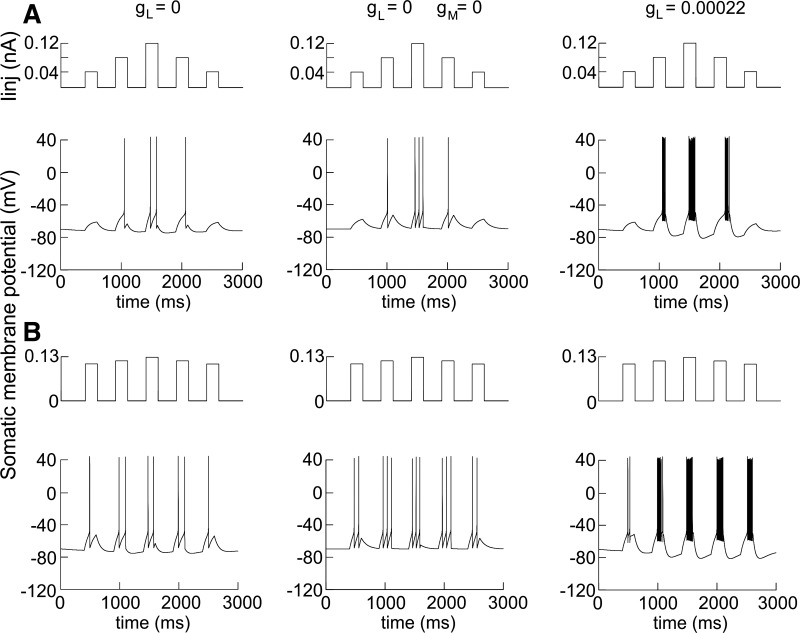
Phasic current injection is insufficient for phase lead induction. *A*: phasic current injection (*I*_inj_) to modeled neuron, applied with linearly increasing/decreasing amplitudes: 0.04 nA (1st and 5th), 0.08 nA (2nd and 4th), and 0.12 nA (3rd injection). *Bottom* panels represent the evoked spiking with high-threshold calcium channel conductance of *g*_L_ = 0 S/cm^2^ (*left*), *g*_L_ = 0 S/cm^2^ and *g*_M_ = 0 S/cm^2^ (*center*), and *g*_L_ = 0.00022 S/cm^2^ (*right*). *B*: phasic current injection (*I*_inj_) to modeled neuron, applied with the following amplitudes: 0.105 nA (1st and 5th), 0.115 nA (2nd and 4th), and 0.125 nA (3rd injection). *Bottom* panels represent the evoked spiking with high-threshold calcium channel conductance of *g*_L_ = 0 S/cm^2^ (*left*), *g*_L_ = 0 S/cm^2^ and *g*_M_ = 0 S/cm^2^ (*center*), and *g*_L_ = 0.00022 S/cm^2^ (*right*).

Strong synaptic input can evoke irregular behavior without being modeled with sinusoidal injection current. Phasic strong synaptic input is sufficient to generate such high frequencies without the presence of intrinsic calcium currents. This raises the fundamental question of whether phasic inputs could explain the firing of thalamic HD neurons in vivo. We addressed this issue by modeling strong phasic inputs without a high-threshold Ca^2+^ current. We applied five constant current injections with a duration of 200 ms each, with linearly increasing (0.04, 0.08, 0.12 nA) and linearly decreasing current values representing the directional tuning curve (Fig. 11*A*, *top*). When *I*_L_ is inactivated (*g*_L_ = 0), there is a formation of spike doublets during 0.12-nA input, with a symmetrical distribution of the output spikes (Fig. 11*A*, *left*; separation angle = 0°). Similarly, concurrent inactivation of *I*_M_ (*g*_M_ = 0) resulted in triplets with a symmetrical output (Fig. 11*A*, *center*; separation angle = 0°). Furthermore, even the reintroduction of *I*_M_ and *I*_L_ (*g*_L_ = 0.00022 S/cm^2^) resulted in a phase lag instead of a phase lead, with a negative separation angle (Fig. 11*A*, *right*; separation angle = −2.15°). Importantly, increasing the current amplitude for all the phasic inputs (0.105, 0.115, and 0.125 nA) also led to symmetrical firing for *g*_L_ = 0 (Fig. 11*B*, *left*) and *g*_L_ = 0, *g*_M_ = 0 (Fig. 11*B*, *center*), with no phase lead (separation angle = 0° for both cases, respectively). Additionally, the reintroduction of *I*_M_ and *I*_L_ resulted in a phase lag with more spikes on the descending slope (Fig. 11*B*, *right*) and negative separation angle (separation angle = −5.26°). These data show that strong phasic current is able to evoke an irregular firing mode, without the incorporation of calcium currents; however, this outcome is paralleled by the absence of phase lead induction.

## DISCUSSION

Here we demonstrate turn-specific modulation of the HD tuning curve and provide evidence that this modulation is a function of the irregular firing patterns of thalamic directional neurons. We use a Hodgkin-Huxley model to show that the spiking phase lead to sinusoidal depolarization input mediates the difference in the tuning curves of CW versus CCW turns. For neurons with adaptive firing properties, the firing frequency is higher on the ascending slope of the injection depolarizing current.

### 

#### Evaluation of turn-specific modulation of directional tuning curve.

To evaluate the turn-specific modulation of directional tuning curves, we used the difference in the mean firing rates for CW and CCW turns, expressed in degrees (Blair and Sharp 1995). The amount of time that turn-specific peak firing (CW or CCW) precedes the averaged HD (for both CW and CW turns) is referred to as the anticipatory time interval (ATI) (Blair et al. 1997). However, the suggestion that ATI implies an active prediction process has been challenged (van der Meer et al. 2007). Because the ATI terminology proposing a process of anticipation might be misleading, we decided to use the separation angle as a measure of the turn-specific modulation of the HD tuning curve. Furthermore, the evaluation of ATI with 60-Hz video monitoring is imprecise, resulting in different values for equivalent studies (Blair et al. 1998; Blair and Sharp 1995; Stackman et al. 2003; Taube and Muller 1998). To avoid errors in the evaluation of the directional signal on a millisecond scale, we measure here the amplitude and the sign of the thalamic separation angle (Taube and Muller 1998). The mean value of the separation angle in our study is 5.96 ± 0.5° for AD nucleus and 8.67° ± 0.6° for AV nucleus. This is consistent with earlier findings from AD thalamus, showing a separation angle value of 6.0 ± 0.9° (Taube and Muller 1998). This approach allowed us to examine differential effect on the formation of separation angle as a result of the AD- and AV-type spiking properties. ISI variability analysis allows us to examine the degree of firing irregularity (Holt et al. 1996; Softky and Koch 1993). HD spike trains within their preferred firing direction are characterized by short ISIs (up to 2 ms), resulting in a low mean firing rate, whereas the ISIs in the periphery of the directional firing range are much longer (>200 ms), resulting in high values of the coefficient of variation > 1. Here we included in the coefficient of variation long ISIs (up to 500 ms) that occur between the spike trains and particularly in the slopes of the tuning curve, which earlier studies excluded (Taube 2010). Using this approach, we investigated the role of the firing patterns in the initiation and the termination of the HD tuning curve (ascending and descending slopes, respectively) in the turn-specific modulation of HD signal and the formation of the separation angle. We show here that the amplitude of the separation angle correlates positively with the degree of ISI variation. To find a causal relationship between rhythmic spike train generation and the degree of separation angle, we used a computational model addressing the role of the major currents that define whether a neuron is irregularly or regularly firing. To confirm or exclude the role of hyperpolarization currents in our model, we recorded HD cells during high-voltage spindle periods and evaluated whether they are entrained by the inhibitory drive of thalamic reticular inputs (Steriade et al. 2001).

#### Anterior thalamic circuitry and head direction activity during spindles.

The ultrastructural organization of anterior thalamus is similar to that of other thalamic nuclei (Somogyi et al. 1978), and anterior thalamic cells express the basic electrophysiological properties (Pare et al. 1987, 1990) that characterize most dorsal thalamic neurons (Jahnsen and Llinas 1984a, 1984b). The current understanding for this region is that the neurons of the AD and AV are relatively homogeneous in morphology and connections. There is little evidence for interneurons in these nuclei; it would appear that majority of AD and AV neurons are projection neurons (Vertes et al. 2001). Concurrently, more than half of all AD neurons (∼60%) are estimated to be HD cells (Taube 1995; Taube and Bassett 2003). Acetylcholinesterase and butyrylcholinesterase (a coregulator of cholinergic neurotransmission) are markers used to differentiate anterior thalamic nuclei (Darvesh et al. 2003). Acetylcholinesterase is found in the highest concentrations in axons in the AD and AV, while butyrylcholinesterase is more concentrated in neurons in AD and latero-dorsal thalamic nucleus (Robertson et al. 1986; Tago et al. 1992). These findings provide additional support for the application of *I*_M_ in the basic Hodgkin-Huxley model for anterior thalamic neurons.

The thalamic reticular nucleus projects GABAergic efferents to rodent anterior thalamus, thus providing the majority of inhibitory inputs to anterior thalamic cells (Wang et al. 1999). One way to test the hypothesis that inhibitory inputs evoke irregular firing patterns of thalamic HD units would be to stimulate or inactivate the thalamic reticular nucleus. However, the connections between rodent reticular and anterior thalamic nuclei are topographically organized (Gonzalo-Ruiz and Lieberman 1995; Lozsadi 1995; but see Pare et al. 1987), and stimulation or inactivation of nontopographical connections might bias the change of individual HD firing rate as a result of network reconfiguration. We used a noninvasive approach by investigating the thalamic HD signal during immobility (particularly the spindle oscillation phase, which is characterized by robust reticular nucleus activity) and comparing the spindle spiking patterns of HD cells to the preceding exploration periods. The activity of thalamic HD cells recorded in behaving animals is updated continuously because of the self-maintaining activity of the tegmento-diencephalic network, also known as the HD attractor network (Blair and Sharp 1995; McNaughton et al. 1991; Redish et al. 1996; Skaggs et al. 1995; Song and Wang 2005). The vestibular system, which contains tonically active neurons even during sleep (Nelson et al. 2003), is proposed to be one of the main sources that generate the HD signal (Stackman and Taube 1997). We compared the HD firing rates of spindle periods and preceding exploration periods. HD spiking during the spindle periods preserved its directionality with a concurrent peak firing rate of ∼60% compared with the preceding exploration periods. This firing decrease was more expressed in neurons with high separation angles (46 ± 10.5%), proposing a role for frequency adaptation currents in HD spiking behavior. While local GABAergic inhibition is not a characteristic network feature of rodent anterior thalamus (Wang et al. 1999), the main GABAergic inputs are provided by the thalamic reticular nucleus (Gonzalo-Ruiz and Lieberman 1995). To determine whether robust reticular activity triggers irregular spike trains in HD cells, we investigated the relation of the HD firing to the phase of high-voltage spindles. Unlike the firing of non-HD units, the firing of HD units was not entrained by thalamic spindle rhythms, suggesting that hyperpolarization currents are not the major mechanism that mediate irregular firing in thalamic HD units.

#### Models of turn-specific modulation in head direction circuitry.

Models that reduce the number of currents involved in spike generation, and yet are still dynamically precise, are either integrate-and-fire models (Brette and Gerstner 2005; Smith et al. 2000) or Hodgkin and Huxley-type models (Foster et al. 1993; Pospischil et al. 2008). In the present paper, we focus on the latter type to simulate the intrinsic properties of thalamic HD neurons (Pospischil et al. 2008). Simplified single-compartment Hodgkin-Huxley-type models were proposed for thalamic cells and derived from more complex models (Destexhe et al. 1996, 1998; Rinzel 1987; Rose and Hindmarsh 1989). We aimed here to model the basic mechanisms that differentiate thalamic spiking patterns into regular and irregular modes, and a single-compartment Hodgkin-Huxley model allowed us to demonstrate the role of main neuronal currents in this process. Previous modeling examining the HD tuning curve properties as a result of dynamic response to head movements have acknowledged the role of medial vestibular nucleus neurons in the formation of ATI in HD circuitry (van der Meer et al. 2007). The model predicted that movement patterns with high-frequency components result in lower anticipation than lower-frequency movements (van der Meer et al. 2007). Although some differences between the biological and modeled data remained unexplained, the authors of this model demonstrated that the firing properties of medial vestibular nuclei neurons afferent to the HD system could be important in the generation of generating HD anticipation. Anatomically, the anterior thalamus receives inputs from the medial vestibular nuclei via the dorsal tegmental nuclei of Gudden (Hayakawa and Zyo 1984) and the lateral mammillary bodies (Gonzalo-Ruiz et al. 1992). Interestingly, the neuronal response in the medial vestibular nuclei expresses a phase lead relative to a sinusoidal angular head velocity input (Kaufman et al. 2000). This firing behavior is likely to be observed in other brain areas including the structures of the HD system, which might be the case with the separation angle/ATI formation in lateral mammillary bodies and AD thalamic nucleus. Experimental results show that ATI is not abolished by passive movements (Bassett et al. 2005), suggesting that ATI might be triggered by intrinsic spike generation currents. Attractor-based models of the HD system suggested a theoretical angular acceleration component (Zhang 1996) or offset connections between HD neurons (Goodridge and Touretzky 2000; Redish et al. 1996) to explain the tuning curve modulation with the effect of angular speed. Our model shows how intrinsic current properties of thalamic HD neurons can generate such turn-specific modulation without requiring specialized neural circuitry. The present model demonstrates that a simple phase shift of the spiking activity to sinusoidal current can generate a separation angle. Phasic injection current implies the alternative scenario for irregular firing within the HD tuning curve. However, such current injection that mimics multiple in-phase synaptic inputs failed to evoke phase lead in our model, regardless of the contribution of high-threshold Ca^2+^ currents. Thus continuous sinusoidal current injection that represents the Gaussian distribution of multiple out-of-phase inputs is the most likely current injection model that explains the formation of the phase lead and positive separation angle of the HD cells. Complex models that take into account not only all anatomical and physiological neuronal properties but also their network connectivity as well as irregular patterns of current injection would recreate more accurately the spiking firing rate of thalamic neurons in vivo.

#### Role of intrinsic currents in phase lead.

Spike rate adaptation and postinhibitory rebound are used to explain ATI formation in modeled vestibular activity (van der Meer et al. 2007). This model explained 60–80% of the behaviorally observed anticipation variability, leaving the possibility for an additional mechanism that regulates this phenomenon. Here we demonstrated that thalamic neurons may be involved in the formation of ATI in the HD system, indirectly represented by the separation angle. We also used spike rate adaptation to generate the separation angle; however, considering the microcircuit anatomy of anterior thalamic nuclei, we modeled excitatory-driven currents to include a spike-triggering mechanism for the dynamic formation of the HD tuning curve. Using a Hodgkin-Huxley-type model we showed here the importance of irregular spike trains in the turn-specific modulation of the directional tuning curve. In light of our spindle data, we deliberately deemphasized the role of low-threshold Ca^2+^ currents in generation of irregular firing by HD thalamic cells. These currents require a preceding hyperpolarization to activate T-type calcium channels (Jahnsen and Llinas 1984b; Zhan et al. 1999), suggesting the leading role of depolarization-triggered L-type Ca^2+^ current for our neurons. We show here that *I*_L_ increases the phase lead to a sinusoidal depolarizing input (Pospischil et al. 2008). Neurons with high L-current conductance (*g*_L_ = 0.00022 S/cm^2^), characterized by a higher ISI coefficient of variation, fire with higher frequency on the ascending slope of the sinusoidal input. As a result, simulations with high *g*_L_ evoke a larger separation angle (7.21°) compared with the moderate-*g*_L_ group (5.40°). Computational models of pyramidal neurons have also demonstrated that bursts occur preferentially on the positive slope of the input signal and this process requires adaptation currents (Kepecs et al. 2002). Here we show that the adaptation current, *I*_M_, is concurrently involved in the phase lead. Neurons without *I*_M_ fire with the highest frequency on the peak of sinusoidal input (with a separation angle close to 0). Our data also reveal that frequency adaptation underlies the effect of angular velocity on directional neurons and this effect is potentiated by depolarization-triggered Ca^2+^ currents. Several Ca^2+^ channel subtypes (N, P, Q, and R types from Ca_v_2 family channels) are also known to contribute to depolarization-triggered currents involved in irregular spike train initiation in vitro (Jung et al. 2001). However, our goal here was to show that a reduced model, embracing multiple subtype depolarization currents into a common high-threshold Ca^2+^ current group (*I*_L_), can elucidate the functional effect of irregular firing on the directional tuning curve.

Here we propose that the separation angle and the relation to the angular velocity of HD neurons across the regions of the HD system might reflect the combination of the spike-triggering excitatory, inhibitory, and adaptation currents. For example, the neurons in postsubiculum contain Ca^2+^-sensitive, nonspecific cation current, *I*_CAN_, that is responsible for the cells' persistent spiking (Yoshida and Hasselmo 2009). However, postsubicular HD cells do not show significant adaptation of the peak firing rate, unlike the AD thalamic HD cells (Taube and Muller 1998). Such a difference might explain why the postsubicular HD neurons express very low values of separation angle compared with the thalamic HD neurons (Taube and Muller 1998). Similarly, the mean correlation between firing rate and angular head velocity (°/s) for postsubicular HD neurons is significantly lower than that for thalamic HD cells (Taube and Muller 1998). Our modeled and experimental data strongly support the idea that the difference of directional turn-specific properties between regions depends on the balance between spike-triggering and adaptation currents of the HD cells.

#### Angular velocity effect mediated by intrinsic spiking properties of thalamic neurons.

The degree of thalamic separation angle is linked to angular velocity in a study showing that the separation angle for fast (>270°/s) turns is 9.79° and that for slow (<270°/s) turns is 4.03° (Blair and Sharp 1995). Concomitantly, under low simulation speed (20°/s) our modeled separation angle for the *g*_L_ = 0 group is 4.53°. Thus our parallel aim was to establish the mechanisms that relate angular velocity to the firing properties of thalamic cells. We confirmed the ability of thalamic cells to fire in relation to the angular velocity. Concurrently, the group of thalamic cells with high separation angle (>8°) is characterized with a higher ISI variability. To find out whether irregular firing determines the dependence on angular velocity, we used a Hodgkin-Huxley-type model addressing the role of the major currents that define whether a neuron is regularly or irregularly spiking. We demonstrate here that the angular velocity effect on neuronal frequency depends on the degree of high-threshold calcium and spike adaptation. Modeled neurons with high *g*_L_ show a higher degree of firing frequency adaptation compared with neurons without high-threshold Ca^2+^ current. Long duration (seconds) in a preferred directional bin for neurons with high *g*_L_ resulted in a lower firing frequency compared with shorter durations, demonstrating the role of velocity (bin/s) in the frequency regulation.

In conclusion, our data present evidence that intrinsic firing properties regulate the encoding of directional information in thalamic networks. Understanding signal processing by hippocampo-diencephalic circuitry will allow us to further develop the models of spatial navigation and path integration.

## GRANTS

This work was supported by Wellcome Trust Grant No. 081075 to J. P. Aggleton, S. M. O'Mara, J. T. Erichsen, and S. D. Vann.

## DISCLOSURES

No conflicts of interest, financial or otherwise, are declared by the author(s).

## AUTHOR CONTRIBUTIONS

Author contributions: M.T. and S.M.O. conception and design of research; M.T. performed experiments; M.T., E.C., M.S.N., and C.E. analyzed data; M.T., R.B.R., J.P.A., J.T.E., and S.D.V. interpreted results of experiments; M.T. prepared figures; M.T. and S.M.O. drafted manuscript; M.T., R.B.R., J.P.A., S.D.V., and S.M.O. edited and revised manuscript; M.T., J.T.E., and S.M.O. approved final version of manuscript.

## Appendix

### Sodium and potassium currents to generate action potentials.

*I*_Na_ is fast voltage-dependent Na^+^ current (kinetics from Traub and Miles 1991):

$$I_{\text{Na}}={g_{\text{Na}}}_{}{\;m}^{3}h\left(V-E_{\text{Na}}\right)$$

$$\text{d}m/\text{d}t=\alpha_{m}\left(V\right)\left(1-m\right)-\beta_{m}\left(V\right)m$$

$$\text{d}h/\text{d}t=\alpha_{h}\left(V\right)\left(1-h\right)-\beta_{h}\left(V\right)h$$

$$\alpha_{m}=-0.32\left(V-VT-13\right)/\exp\left[-\left(V-VT-13\right)/4\right]-1$$

$$\beta_{m}=0.28\left(V-VT-40\right)/\exp\left[\left(V-VT-40\right)/5\right]-1$$

$$\alpha_{h}=0.128\exp\left[-\left(V-VT-17\right)/18\right]$$

$$\beta_{h}=4/1+\exp\left[-\left(V-VT-40\right)/5\right]$$

where *g*_Na_ = 50 mS/cm^2^ and *E*_Na_ = 50 mV.

*I*_Kd_ is “delayed-rectifier” K^+^ current (Traub and Miles 1991):

$$I_{\text{Kd}}=g_{\text{Kd}}n^{4}\left(V-E_{\text{K}}\right)$$

$$\text{d}n/\text{d}t=\alpha_{n}\left(V\right)\left(1-n\right)-\beta_{n}\left(V\right)n$$

$$\alpha_{n}=-0.032\left(V-VT-15\right)/\exp\left[-\left(V-VT-15\right)/5\right]-1$$

$$\beta_{n}=0.5\;\exp\left[-\left(V-VT-10\right)/40\right]$$

where *g*_Kd_ = 5 mS/cm^2^ and *E*_K_ = −90 mV.

### Slow potassium current for spike-frequency adaptation.

*I*_M_ is a slow noninactivating K^+^ current (kinetics from Yamada et al. 1989):

$$I_{\text{M}}=g_{\text{M}}\;p\left(V-E_{\text{K}}\right)$$

$$\text{d}p/\text{d}t=\left[p\infty \left(V\right)-p\right]/\text{τ}\tau_{p}\left(V\right)$$

p∞(V)=1/1+exp[−(V+35)/10]

$$\tau_{p}\left(V\right)=\tau_{\max}/3.3\;\exp\left[\left(V+35\right)/20\right]+\exp\left[-\left(V+35\right)/20\right]$$

where *g*_M_ is 0.004 mS/cm^2^ and τ_max_ = 4 s, unless stated otherwise.

### Calcium current to generate irregular spike trains.

*I*_L_ is L-type Ca^2+^ current (kinetics from Reuveni et al. 1993):

$$I_{\text{L}}=g_{\text{L}}q^{2}r\left(V-E_{\text{Ca}}\right)$$

$$\text{d}q/\text{d}t=\alpha_{q}\left(V\right)\left(1-q\right)-\beta_{q}\left(V\right)q$$

$$\text{d}r/\text{d}t=\alpha_{r}\left(V\right)\left(1-r\right)-\beta_{r}\left(V\right)r$$

$${\text{α}}_{q}=0.055\left(-27-V\right)/\exp\left[\left(-27-V\right)/3.8\right]-1$$

$${\text{β}}_{q}=0.94\exp\left[\left(-75-V\right)/17\right]$$

$${\text{α}}_{r}=0.000457\exp\left[\left(-13-V\right)/50\right]$$

$${\text{β}}_{r}=0.0065/\exp\left[\left(-15-V\right)/28\right]+1$$

where *g*_L_ is the maximum conductance of *I*_L_ and the reversal potential for Ca^2+^ ions is *E*_Ca_ = 120 mV.
